# Recent Advances in Nanocellulose Aerogels for Efficient Heavy Metal and Dye Removal

**DOI:** 10.3390/gels9050416

**Published:** 2023-05-16

**Authors:** Azfaralariff Ahmad, Mohamad Anuar Kamaruddin, Abdul Khalil H.P.S., Esam Bashir Yahya, Syaifullah Muhammad, Samsul Rizal, Mardiana Idayu Ahmad, Indra Surya, C. K. Abdullah

**Affiliations:** 1 Bioresource Technology Division, School of Industrial Technology, Universiti Sains Malaysia, Penang 11800, Malaysia; 2 Green Biopolymer, Coatings and Packaging Cluster, School of Industrial Technology, Universiti Sains Malaysia, Penang 11800, Malaysia; 3 Environmental Technology Division, School of Industrial Technology, Universiti Sains Malaysia, Penang 11800, Malaysia; 4 Bioprocess Technology Division, School of Industrial Technology, Universiti Sains Malaysia, Penang 11800, Malaysia; 5 Chemical Engineering Department, Universitas Syiah Kuala, Banda Aceh 23111, Indonesia; 6 ARC-PUIPT Nilam Aceh, Universitas Syiah Kuala, Banda Aceh 23111, Indonesia; 7 Mechanical Engineering Department, Universitas Syiah Kuala, Banda Aceh 23111, Indonesia; 8 Renewable Biomass Transformation Cluster, School of Industrial Technology, Universiti Sains Malaysia, Penang 11800, Malaysia; 9 Department of Chemical Engineering, Universitas Sumatera Utara, Medan 20155, Indonesia

**Keywords:** aerogel, cellulose, pollutant, dyes, heavy metals, water treatment

## Abstract

Water pollution is a significant environmental issue that has emerged because of industrial and economic growth. Human activities such as industrial, agricultural, and technological practices have increased the levels of pollutants in the environment, causing harm to both the environment and public health. Dyes and heavy metals are major contributors to water pollution. Organic dyes are a major concern because of their stability in water and their potential to absorb sunlight, increasing the temperature and disrupting the ecological balance. The presence of heavy metals in the production of textile dyes adds to the toxicity of the wastewater. Heavy metals are a global issue that can harm both human health and the environment and are mainly caused by urbanization and industrialization. To address this issue, researchers have focused on developing effective water treatment procedures, including adsorption, precipitation, and filtration. Among these methods, adsorption is a simple, efficient, and cheap method for removing organic dyes from water. Aerogels have shown potential as a promising adsorbent material because of their low density, high porosity, high surface area, low thermal and electrical conductivity, and ability to respond to external stimuli. Biomaterials such as cellulose, starch, chitosan, chitin, carrageenan, and graphene have been extensively studied for the production of sustainable aerogels for water treatment. Cellulose, which is abundant in nature, has received significant attention in recent years. This review highlights the potential of cellulose-based aerogels as a sustainable and efficient material for removing dyes and heavy metals from water during the treatment process.

## 1. Introduction

The environment is threatened by pollution because of rapid technological advancements and the continuous growth of daily supply demands and necessities. Over the past two decades, industrial and agricultural growth has polluted waterways and landfills, evidenced by the environmental stress shown by water quantity and quality. Climate change, population growth, rising standards of living, and uneven water distribution are directly related to competition for water resources, water scarcity, poor waste quality, and hydrological disasters [1]. In addition, approximately 300–500 million tons of wastewater are dumped into the environment annually from various sectors, including industries and agriculture [2]. Textile industries, paper industries, metal plating industries, fertilizer industries, agricultural waste, and mining activities are the largest contributors to water pollution. There are various types of pollutants, including organic dyes, phenols, biphenyls, pesticides, fertilizers, hydrocarbons, plasticizers, detergents, oils, greases, medicines, proteins, carbohydrates, etc. [3]. Even if most toxic chemical wastes are filtered before discharge, leakages into the environment seem unavoidable, posing substantial problems and often affecting global health because these chemicals can be stored by organisms in the environment and biomagnified through food chains. Water pollutants can be classified into organic and inorganic pollutants. Carcinogenic organic contaminants directly affect human health, while inorganic nutrients such as nitrogen or phosphorus cause waterbodies to eutrophicate and harm aquatic life [2,4]. In addition, organic dyes in water can absorb sunlight and increase the temperature, thus affecting the growth of aquatic bacterial species and disrupting the ecological balance. Organic dyes are more harmful than other contaminants because they are chemically stable and do not easily degrade in water [2,5]. With more than 100,000 commercially available dyes, mostly used in textile and food industries, over 7 × 10^5^ tons is produced annually in water [2]. Effective water treatment procedures can prevent such environmental damage. In recent years, several studies have focused on organic dye removal from water. Adsorption, precipitation, and filtration are the main water treatment procedures [6]. Adsorption is simple, efficient, and cheap considering its materials and operation. The target compound accumulates on an adsorbent’s porous surface, which has a high surface area. Among the adsorbent materials studied, the use of aerogels is growing exponentially. Aerogels are freeze-dried precursor hydrogel systems with low density and high porosity. Depending on the material used, they have a soft and flexible nature, a high surface area, and low thermal and electrical conductivity and can respond to external stimuli [2]. They can absorb several wastewater toxins in a simple, sustainable, low-cost, and safe way. According to Abdul Khalil and colleagues, biomaterials have emerged as the leading option for producing aerogels because of their widespread availability, abundance, low cost, and renewability [7]. Biopolymers such as cellulose, starch, chitosan, chitin, carrageenan, and graphene are being extensively researched to develop sustainable aerogels for water treatment. Biomaterials are the primary choice in the production of aerogels because of their availability, abundance, low cost, and renewability. Sustainable aerogels for water treatment use biopolymers such as cellulose, starch, chitosan, chitin, carrageenan, and graphene, which have been extensively studied. Out of these, cellulose has gained significant attention in recent years as it is the most abundant biopolymer from plants found in nature. This review highlights the potential of nanocellulose-based aerogels as a sustainable and efficient material for removing dyes and heavy metals from water during the treatment process.

## 2. Nanocellulose-Based Aerogels as Functional Materials

Aerogels are a fascinating class of solid materials that have a unique porous structure, making them incredibly lightweight. With high porosity ranging from 80% to 99.8%, aerogels exhibit a very low bulk density of 3–150 mg/cm^3^ and a large surface area (500–1000 m^2^/g) [2]. They are synthesized using a sol–gel process and drying technology on organic, inorganic, or hybrid molecular precursors, which results in a solid material with exceptional properties such as low electric resistivity, changeable surface chemistry, low thermal conductivity, large surface area, and high hydrophobicity. The name “aerogel” comes from the fact that air replaces the liquid without changing the solid’s microstructure. Studies have advanced from SiO_2_ aerogels, and aerogels have been created from a range of materials including inorganic (such as SiO_2_ derived from various alkoxysilanes: TiO_2_, Al_2_O_3_, ZrO_2_, etc.), organic (such as resorcinol–formaldehyde, polyurethane, polyimide, polystyrene, etc.), carbon (such as carbon, carbon nanotubes, graphene), semiconductor chalcogenides (CdS, CdSe, PbTe), natural materials (such as cellulose and other polysaccharides and proteins), and others [2].

### 2.1. Nanocellulose as a Precursor Material

**Figure 1:**
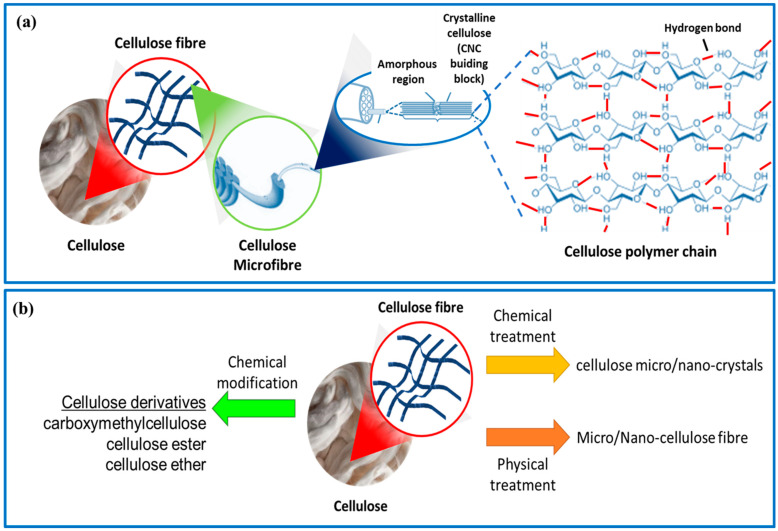
(**a**) Morphology illustration of plant cellulose fibers and their molecular structure. (**b**) Schematic of the preparation of cellulose micro/nano-crystals/fibers and derivatives.

Cellulose is the most abundant natural polysaccharide and a major structural component in wood, cotton, natural fibers, and the lignocellulosic components of plants. Annually, it is estimated that approximately 1 × 10^12^ tons of the total biomass produced is cellulose [8], making it the most exploitable functional material among natural polysaccharides. Cellulose is a biopolymer made up of repeating units of anhydrous glucose (AGU) linked by acetal bonds and hydroxyl (OH) groups along the macromolecule chain (Figure 1a). Cellulose fibers exhibit a unique structural hierarchy derived from their biological origin. It can be extracted from a wide range of different sources, which mainly include plant-based materials and plant parts such as the stem, bark, leaves, fruit rice straws, kenaf, cotton, wood, potato tubers, and bagasse [9]. Cellulose can also be produced from microbes and sea animals in larger quantities, such as bacteria, algae, fungi, and tunicates [10]. It is composed of assemblies of microfibrils (Figure 1a), which form slender and nearly endless rods in the cell walls of plants, fungi, algae, and bacteria, providing structural support [11]. The properties of cellulose, such as molecular chain length, size, degree of crystallinity, and thermal stability, depend on the plant species and extraction methods used, including pre-treatment, post-treatment, and disintegration processes [12,13]. Through acid and/or enzymatic hydrolysis or physical treatment, these microfibrils can break down into short crystalline rods or ‘‘cellulose micro/nano-crystals/fibers” (Figure 1a). In addition, cellulose can also be produced through the static cultivation of bacteria such as Acetobacter xylinum. The chemical structure of bacterial cellulose is similar to that of plant cellulose, but it has a higher degree of crystallinity (>80%) and lacks impurities such as lignin and hemicellulose. The physical and biological properties of bacterial cellulose are superior to those of plant cellulose [14].

Cellulose is a popular choice for sustainable materials because of its abundant, renewable, and biodegradable nature [15,16]. Each glucose unit in the cellulose chain contains three hydroxyl groups, two secondary alcohols in C2 and C3, and one primary alcohol in C6, which are highly reactive and can be easily modified [17]. This results in a variety of cellulose derivatives (Figure 1b) that can be created through chemical reactions such as oxidation, esterification, etherification, acylation, grafting copolymerization, and crosslinking reactions [18,19]. Some cellulose derivatives include carboxymethylcellulose, cellulose ester, and cellulose ether, which can be created through processes such as grafting, sulfonation, and TEMPO-mediated oxidation [20]. There are numerous reviews available that discuss the structure, properties, and applications of cellulose and its derivatives.

### 2.2. Nanocellulose Aerogels

Aerogels are materials made by using a sol–gel process and drying technology on organic, inorganic, or hybrid molecular precursors, resulting in a solid material with high surface area, low density, low electric resistivity, and changeable surface chemistry [2]. An aerogel can be classified according to its drying process (aerogel, xerogel, and cryogels), surface functionality (hydrophilic, hydrophobic, amphiphilic, oleophilic, or oleophobic.), and the type of precursor (organic, inorganic, or hybrid). The first aerogels were created by S. Kistler in 1931 using silicon dioxide (SiO_2_) and supercritical drying to remove the solvent from the pores of wet gels [21,22]. The small spherical SiO_2_ clusters (3–4 nm in diameter) in aerogels form chains and a grid with air-filled pores, giving them excellent thermal insulation properties (conductivity lower than 28 mWm^−1^·K^−1^) because of a small pore size that limits gas phase conduction [23]. In the past, low thermal conductivity was the most researched aspect of aerogels, but because of their small pore size and high surface areas, their use is now being explored for various other applications, such as removing water pollutants, drug delivery [24], sensors [25], gas absorption [26], radioactive waste confinement [27], nuclear particle detection [28], electronic devices [29], catalysis [30], and surface coatings (self-cleaning coatings and chemicals) [31]. Depending on the purpose and the production method, aerogels can be produced either in monolith or particle form. Despite their potential, these materials have limitations, such as fragility, stiffness, dust generation, and bulkiness, preventing widespread use. To overcome these issues, various approaches have been explored, including changing the material used and improving preparation techniques to enhance their properties.

### 2.3. Fabrication Stages of Aerogels

**Figure 2:**
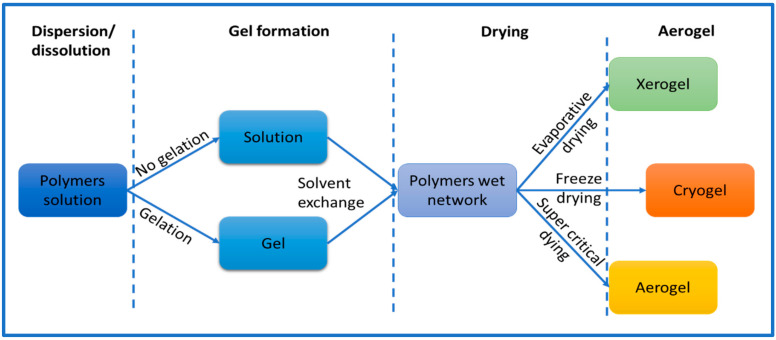
Common approach to preparing aerogels.

Cellulose aerogel is a type of solid that has a porous structure, and it is made by using materials that are derived from cellulose. Researchers have been exploring different ways to produce aerogels by using cellulose and its derivatives. Generally, the process of making cellulose aerogel involves three stages (Figure 2). First, the materials are either dissolved or dispersed in a liquid. Then, a gel is formed by allowing the liquid to solidify. Finally, the gel is dried to create the aerogel. To produce nanocellulose aerogel, the standard procedure involves dispersing nanocellulose in solvent using either ultrasonic or mechanical methods [9]. The initial stage of the process requires selected cellulose fibers to be dispersed in a suitable solvent, such as water, alcohol, calcium thiocyanate, NaOH-water solution, or ionic liquids. The selection of a solvent plays a critical role in determining the solubility and gelation of cellulose in the preparation of aerogels. Shen and his colleagues have provided a comprehensive list of solvent mixtures used in the preparation of aerogels. These solvent systems include lithium chloride and dimethylacetamide (LiCl/DMAc), lithium chloride and N-methyl-2-pyrolidone (LiCl/NMP), calcium chloride dihydrate and methanol (CaCl_2_·2H_2_O/MeOH), N-methylmorpholine oxide (NMMO), paraformaldehyde and dimethylsulfoxide (PF/DMSO), triethylammonium chloride and dimethylsulfoxide (TEAC/DMSO), tetrabutylammonium fluoride and dimethylsulfoxide (TBAF/DMSO), and alkali/urea (or thiourea) aqueous systems [32]. The choice of solvent can significantly impact the properties of the final product. The process and materials used during the creation of cellulose aerogels can also affect their properties. For instance, factors such as the size of the particles, the type of cellulose utilized, and the production method can all influence the aerogel’s characteristics, including its porosity, strength, and density. Thus, it is essential to carefully consider all these factors during the production of cellulose aerogels to obtain the desired properties for specific applications.

#### 2.3.1. Gelling Stage

Once a cellulose suspension or solution is prepared, it can be transformed from a suspension or solution into a solid gel in several ways [32,33]. The formation of a stable 3D network structure in a solid gel is crucial for holding a significant amount of water in its interstitial spaces. This can be achieved by crosslinking the polymer chains that make up the gel. In the case of cellulose, it has hydrophilic functional groups, such as hydroxyl (-OH), that enable it to form both physical crosslinking through electrostatic interactions and chemical crosslinking through covalent interactions using a crosslinker.

Physical hydrogels are formed by the crosslinking of polymer chains through physical interactions, including entanglement, van der Waals forces, hydrogen bonds, and hydrophobic or electronic interactions. There are different methods for achieving physical cellulose gels. One straightforward method is to freeze the cellulose solution, which can cause the water to form ice crystals that serve as physical crosslinks. Another method is to use coagulative regeneration, where the cellulose solution is added to a non-solvent, causing the cellulose to dehydrate and form a gel. Another option is the agglomeration process, which involves the addition of a crosslinking agent to the cellulose solution, which causes the formation of a gel.

**Figure 3:**
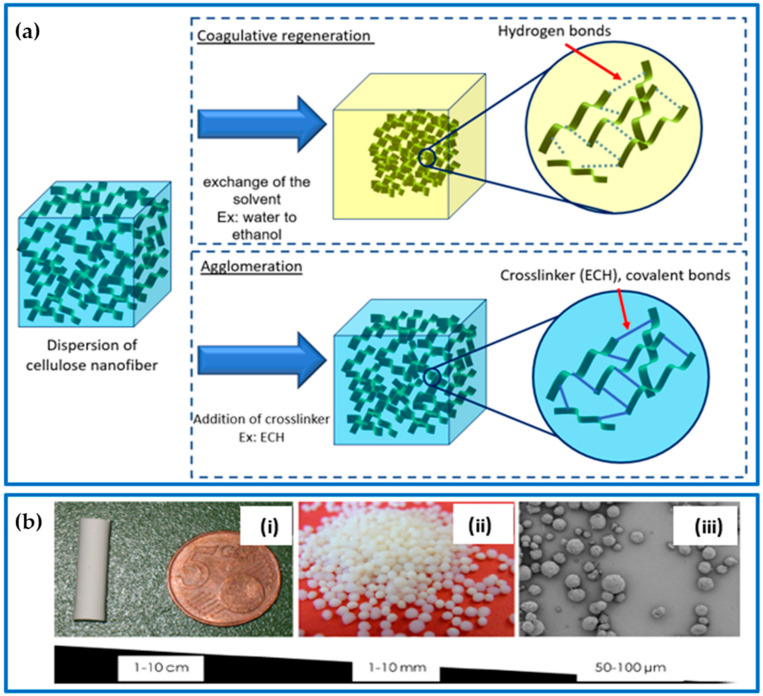
(**a**) Cellulose aerogel gelling approach: coagulative regeneration and chemical agglomeration. (**b**) Different forms of cellulose aerogels: (**i**) monolith, (**ii**) beads, and (**iii**) microparticles; adapted from [39,40,41].

Coagulative regeneration is a widely used technique for creating hydrogels. In this process, a solution containing a polymer is transformed into a gel by carefully removing the solvent [34]. This technique is achieved through the exchange of the solvent with a non-solvent, leading to the desolvation of cellulose molecules and the reformation of hydrogen bonds, which results in the coagulation of the polymer [35]. During this process, the solvent is replaced with a non-solvent, which causes the cellulose molecules to lose their solvents and form bonds via physical crosslinking. The coagulation process can occur in different ways, such as through nucleation/growth or spinodal decomposition. During nucleation/growth, one phase grows in the mixture, while spinodal decomposition results in a periodic variation in concentration, causing phase separation [9]. As the concentration of biopolymers increases, the transformation from a solution to a liquid crystalline gel and then a solid gel with an anisotropic structure occurs (Figure 3a). The stability and organization of the gel structure improve with an increase in the biopolymer concentration because of a higher degree of chain entanglements and increased hydrogen bonding interactions. Electrolytes, such as calcium chloride, can enhance the physical gelation process by impacting the distribution of charges within the solution [36,37]. Similarly, graphene oxide (GO) can interact with cellulose through hydrogen bonds and, therefore, hasten the process of physical gelation as well [38].

Coagulative regeneration is a valuable technique for creating hydrogels with desirable properties. The careful manipulation of the solvent and other variables, such as the biopolymer concentration and the addition of electrolytes or graphene oxide, can lead to the creation of solid gels with specific characteristics that are suitable for various applications.

#### 2.3.2. Aging Stage

Agglomeration is the key aspect involved in the formation of physical hydrogels, where polymer chains are crosslinked through various physical interactions such as entanglement, van der Waals forces, hydrogen bonds, covalent bonds, and hydrophobic or electronic interactions. It can be achieved either physically or chemically. Physical agglomeration involves stirring cellulose particles in a liquid to clump them together, creating larger particles. Chemical agglomeration, on the other hand, involves the addition of crosslinking agents or changes in temperature or pH to create a 3D structure via chemical crosslinking. Physical gels rely on physical interactions, such as van der Waals forces and hydrogen bonds, while chemical gels have covalent bonds [42,43]. The physical approach produces solid gels with unique properties because their network junctions can be created and destroyed by the movement of polymers. The degree and speed of phase separation depend on the type and concentration of the anti-solvent and the temperature used.

Chemical crosslinking is preferred when high mechanical strength is required and is accomplished by introducing a chemical crosslinking agent, forming strong molecular bonds such as via covalent and electrostatic interactions. To make cellulose hydrogels stable and effectively swollen, a 3D hydrophilic network is usually needed, which is achieved by using chemical crosslinkers during the gelation process. In chemical crosslinking, a bond is formed between the crosslinking agent and polymer or between the functional groups of polymer molecules. Cellulose and its derivatives have hydroxyl groups that are connected by crosslinker with an aldehyde group (aldol formation) via a covalent bond. A typical crosslinker for cellulose gels includes glutaraldehyde and epichlorohydrin, N,N0-methylenebisacrylamide (MBA), formaldehyde, and acetaldehyde because of their availability and cost-effectiveness. This chemical will form covalent bonds between the polymer chains, transforming the material into a solid gel [44]. Chemical crosslinkers can be divided into two types: esterifying agents, such as carboxylic acids and anhydrides, and etherifying agents, such as organochlorine, epoxide, and vinyl compounds. The first type creates -COOR bonds while the second type creates R-O-R bonds [44,45].

#### 2.3.3. Shaping of Aerogels

The process used to dissolve or disperse cellulose fibers can affect the structure of the resulting gel. Gel formation is crucial as it determines the network and shape of the pores in the aerogel. Before the final drying process, depending on the purpose and the production method, aerogels can be produced in either monolith or particle forms (Figure 3b). Aerogels can be formed in various sizes and shapes using a variety of techniques such as molding, extrusion, or other physical methods. For instance, molding involves pouring a solution of polysaccharides into a mold with a desired shape, which then undergoes gelation. This shape is retained in the final aerogel after drying. Polysaccharide-based aerogels typically take the form of cylindrical monoliths, but other shapes, such as spheres, tubes, and membranes, can also be produced. Monolithic aerogels are particularly advantageous because of their ease of handling, reusability, and ease of recollection. These materials can be easily manipulated into the desired shapes and sizes, making them ideal for a wide range of applications. Regardless of the shape, aerogels provide a large surface area per unit volume, which is particularly advantageous in various fields such as pharmaceuticals and environmental applications, where fast absorption or dissolution is necessary [41]. 

Aerogels, because of their unique porous structure, already have a high surface area per unit volume. However, their absorption capacity can be further increased by reducing the size of the aerogel particles, which leads to an even larger surface area. One way to achieve this is through particle micronization, such as by milling a monolith aerogel into beads or microspheres. However, this method has some drawbacks, including irregular particle shapes and inconsistent particle sizes. Alternatively, uniform sizes and shapes can be obtained by producing aerogels in the form of beads (millimeter to centimeter range). Aerogels in spherical forms are commonly prepared via droplet forming, gelation, solvent exchange, and suitable drying [32]. The droplets are obtained by dropping the aerogel precursor using a syringe/nozzle into the gelling promoter agent solution (e.g., pH- and/or temperature-controlled solution, cations). Smaller beads and microspherical aerogels in the size range of 1–3000 µm can be obtained by vigorously stirring an aqueous solution (sol dispersed phase) in an oil (continuous phase) emulsion for the purpose of gelling the dispersed phase in tandem with aerogel technology [32]. After gelation is complete, the liquid phase can be replaced with air using freeze-drying or supercritical CO_2_ drying methods.

#### 2.3.4. Drying Process

Drying is the process by which liquid from within the gel is removed without causing substantial damage to the gel’s microstructure; multiple drying procedures are able to be performed based on the type of spherical gel and its mechanical stability [32]. The appropriate drying technologies are the most important step in the fabrication of aerogels and can be accomplished in a number of ways, the most common of which are evaporative drying, freeze-drying, and supercritical drying, which will result in the formation of xerogels, cryogels, and aerogels, respectively. Among these three, aerogels have the largest pore structure, followed by cryogels and xerogels [46]. 

**Figure 4:**
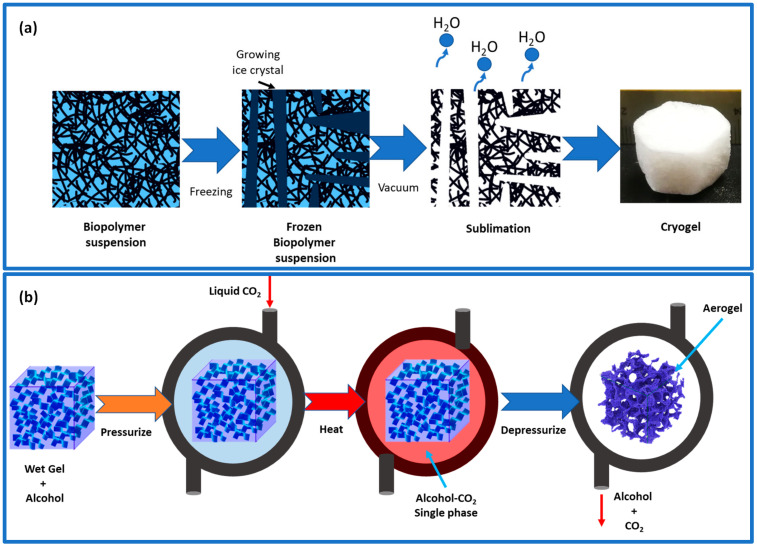
(**a**) Illustration of freeze-casting–freeze-drying principles. (**b**) Illustration of supercritical drying process using a two-component CO_2_–alcohol.

The evaporation process of aerogel can lead to the contraction of its pores because of high temperatures and capillary pressures. To prevent this, a solvent exchange can be performed prior to evaporation, reducing the impact of capillary pressures and reducing the contraction of the pores. Additionally, adding hydrophobic groups to the aerogel’s surface can prevent further shrinking [47]. The presence of these hydrophobic groups can provide a spring-back effect, reducing the influence of capillary forces. This is because hydrophobic groups repel each other, thus reducing contact between them and the polar solvent. Typically, the liquid in hydrogel can be removed using the freeze-drying method, which involves two steps: freezing the liquid samples down below their glass transition or melting point and then eliminating the solvents through sublimation (Figure 4a) [32]. The initial step determines the shape and size of the resulting beads or microspheres, while the final process controls the porous and connected structures. Most solutes and suspended particles in water are separated from the forming ice crystals into the interstitial zone and result in aerogels with porosities similar to ice crystals [48]. The freezing process may cause changes in ice crystal nucleation and growth rates, leading to different pore architectures in the aerogel at different depths. During the natural sublimation of ice crystals during freeze-drying, the organic gel structure may expand, creating macropores and reducing the specific surface area. Another method is freeze-casting, where the liquid is deliberately frozen in one direction by adhering to a pre-set heat gradient [23]. The hydrogel is solidified with the purpose of having the pores form in a specific direction (a 1D, 2D, or radial direction), unlike traditional freezing, where the crystal formation may be random. Once ice crystals have formed, particles are firmly held together by strong hydrogen bonds and van der Waals forces. With this technology, hydrogels with a controlled pore design can be dried, effectively removing a significant portion of mesopores [47].

Supercritical fluid drying is the most popular drying technique; thus, the best way to protect the porous network is to carry out the solvent removal phase using this technique at high temperatures and pressures. Before the supercritical drying of gels that contain water as a solvent, the solvent exchange must proceed since water and CO_2_ cannot mix. The water will be exchanged either with acetone or alcohol because they are very miscible with CO_2_ [32]. Commonly, CO_2_ is used in place of an organic solvent to keep temperatures from rising too high. CO_2_ has a lower processing temperature than an organic solvent because of its low surface tension and lower critical temperature of 31 °C [47].

The supercritical drying process involves a two-way transfer of supercritical CO_2_ and gel solvent in and out of the wet gel’s pores (Figure 4b). A high dissolution of CO_2_ in the gel solvent affects the drying process, leading to increased liquid and the escape of excess liquid from the gel network [32]. When CO_2_ reaches supercritical conditions in the pores, the nanostructure remains intact because of the absence of a vapor–liquid phase and capillary forces. Freeze-drying can also achieve this result if the gel structure is strong enough. Various factors such as time, temperature, pressure, and the depressurization rate determine the final aerogel’s properties, such as porosity, density, and pore structure. Supercritical drying results in high-quality aerogels, but it is a costly and demanding process as it operates at high pressure and requires a regular CO_2_ supply [47].

### 2.4. Modification of Aerogel

The properties and functionalities of aerogel can be further enhanced by modifying their formulation through various methods, including the addition of different materials or changing the concentration of existing components. Such modifications have resulted in the production of aerogels with an improved adsorption capacity for various water pollutants, including oil, dyes, and heavy metals. 

To increase the absorption capability of cellulose aerogels for oil, researchers have modified them with various chemicals, such as methyltrimethoxysilane (MTMS) [49,50], Trimethylchlorosilane (TMCS) [51], Methyltriethoxysilane (MTES) [52], Methyltrichlorosilane (MTCS) [53], Perfluorooctyltriethoxysilane (PFTS) [54], sodium dodecylsulfate (SDS) [55], 1,4-butanediol diglycidyl ether [56], and methylene diphenyl diisocyanate (MDI) [57]. Most of these chemicals have been employed as substitutes for nucleophilic groups such as alcohols, carboxylic acids, and amines by incorporating silyl groups (R3Si-). The process of silylation has been utilized to graft molecules onto cellulose and its derivatives through covalent bonds [58]. As a result of these modifications, cellulose aerogels with significantly improved hydrophobic properties have been produced. This enhanced hydrophobicity renders cellulose aerogels particularly well suited for applications involving the separation of oil and water. By utilizing silylation to introduce these covalent bonds, cellulose aerogels acquire desirable characteristics that enable effective and efficient oil–water separation, providing a promising solution in industries where such separation processes are vital.

In addition to oil, cellulose aerogels have been modified to increase their adsorption capability against other major water pollutants such as dyes and heavy metals. Researchers have modified or blended cellulose aerogels with other components to enhance their functionality in removing dyes and heavy metal contaminants from water. One approach involves blending activated carbon into the formulation, which has been shown to increase the adsorption capacity of cellulose aerogel for dyes and heavy metals such as methylene blue, Rhodamine B, Cu(II), Cd(II), and Hg(II) [59,60]. Graphene and carbon nanotubes have also been used as additives to achieve similar effects [61]. Another approach involves crosslinking cellulose aerogels with polyethyleneimine (PEI) using 3-glycidyloxypropyl trimethoxysilane (GPTMS), which has been shown to increase the adsorption capacity for methylene blue and Pb (II) ions [62]. Other compounds, such as 3-aminopropyltrimethoxysilane (APTMS), N-methylene phosphonic acid (NPCS), and zeolitic imidazolate framework-8 (ZIF-8), have also been reported to increase the adsorption capability of cellulose aerogels against various dyes and heavy metals [63,64].

## 3. Impact of Water Pollutants on Human Health

**Figure 5:**
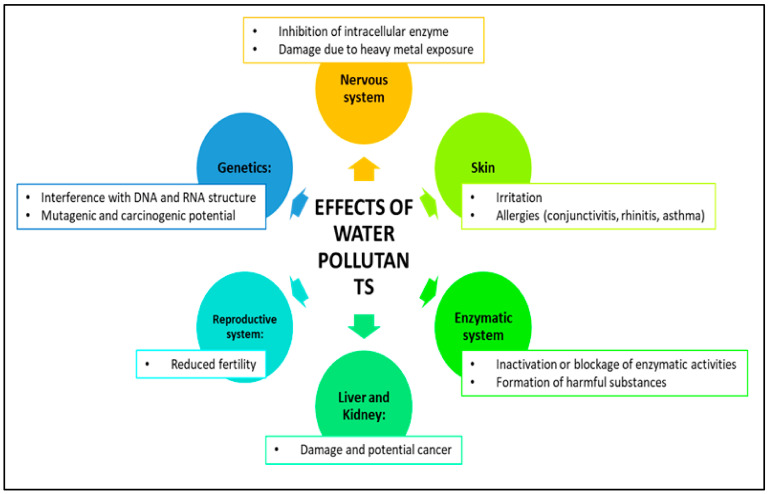
Illustration of the health effects of water pollutants on human health.

Water pollution is a significant problem caused by human activities such as factories, farming, and technology, and it can be harmful to both the environment and people’s health [65]. Pollutants come from various sources, including heavy industries, electronics, textiles, food production, housing, pharmaceuticals, hospitals, mining, and agriculture (Figure 5). These pollutants can be organic or inorganic and can cause health problems such as skin irritation and respiratory issues. They can also disrupt ecosystems, harm animals and humans, and cause chronic conditions [2,66]. Exposure to pollutants can have long-term negative effects on individuals and communities. Water pollution can also reduce biodiversity, negatively impact fishing and agriculture, and make drinking water unsafe. To protect the health of all living beings and our planet’s sustainability, it is essential to take measures to reduce the levels of pollutants in the environment. Dyes and heavy metal pollution in drinking water sources pose a major threat to public health and the environment. These pollutants can be toxic, cause cancer, and upset the natural balance of microbial populations in water, causing serious ecological damage [66]. Inefficient water treatment can release pollutants that harm the environment and living organisms. In developing countries, farmers may use wastewater to irrigate crops, negatively affecting soil quality and crop growth. These chemicals can build up in organisms and enter the human body (Figure 5) through food, water, air, and the skin, causing various health issues with prolonged exposure [67]. To address this issue, researchers are developing new methods and materials to effectively remove these harmful pollutants from water sources. This ongoing research is crucial to safeguarding the health of communities and ensuring the sustainability of our planet.

### 3.1. Impact of Dyes on Human Health

Textile industries release large amounts of colored wastewater containing harmful organic pollutants, mainly azo dyes, which are persistent and hazardous to human health. The use of up to 8000 chemicals and huge amounts of water in the industry leads to the production of highly contaminated wastewater that contains 72 toxic chemicals, with 30 of them unable to be removed with waste treatment methods [4,67,68]. These include substances such as sulfur; naphthol; nitrates; acetic acid; soaps; enzymes; chromium compounds; and heavy metals such as copper, arsenic, lead, cadmium, mercury, nickel, cobalt, and certain auxiliary chemicals [67]. Inefficient dyeing processes and the discharge of untreated wastewater into water resources cause serious ecological damage and toxic effects on living organisms [69]. Exposure to these chemicals can lead to various health issues, ranging from skin irritation to central nervous system disorders. For example, benzidine, a well-known carcinogen, is present in most azo dyes and can cause bladder cancer [5]. Handling reactive dyes can pose a risk to workers, who may develop allergic reactions such as contact dermatitis, allergic conjunctivitis, rhinitis, or occupational asthma [70]. Table 1 lists the common dyes used in the textile industry and their reported health risks.

### 3.2. Impact of Heavy Metals on Human Health

In recent years, the presence of heavy metals in water has become a major global concern. In addition to dyes, the presence of heavy metals required for textile dye pigment production also has toxic effects [82]. These pollutants can have detrimental effects on both human health and natural resources [83]. Heavy metals are harmful because they do not break down easily and can build up in the environment. They can get into water from natural processes such as soil erosion and from human activities such as waste disposal [65]. Urbanization and industrialization have contributed to the increase in heavy metal pollutants in water. There are 35 heavy metals, with lead (Pb), mercury (Hg), arsenic (As), and cadmium (Cd) being the most common pollutants. Copper (Cu), zinc (Zn), and chromium (Cr) are also heavy metals that can be toxic at high levels, although they are necessary in small amounts [65,84]. The effects of these heavy metal pollutants on human health and sources of contamination are summarized in Table 2. The toxicity of these elements is determined by factors such as the type of metal and its biological role in the life cycle. Heavy metal pollutants in water can accumulate in various tissues such as bones, fat, muscle, and joints, leading to disorders including stunted organ growth, cancer, neurological damage, impaired cognitive development in children, damage to the nervous system, kidney damage, and even death (Rachna et al., 2021). It is important that steps are taken to reduce and prevent pollution in water caused by heavy metals in order to protect human health and the environment.

## 4. Application of Nanocellulose Aerogels in Water Treatment

**Figure 6:**
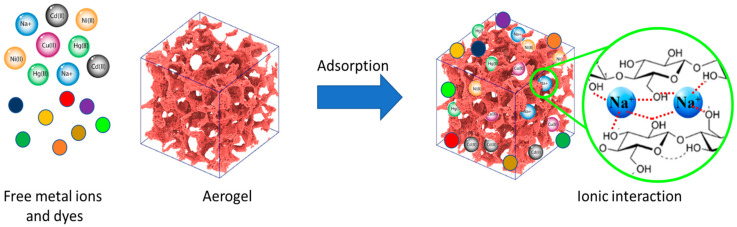
Schematic overview of the adsorption of dyes and heavy metal ions by a cellulose-based aerogel.

Water pollution caused by improper disposal of organic and inorganic contaminants is a major environmental challenge that can lead to long-term harm to the aquatic environment and humans. Dyes, phenolic compounds, metallic ions, and micropollutants such as pesticides and drugs have been found in wastewater, surface water, and even drinking water [91]. Adsorption is one of the most promising techniques for removing these pollutants from wastewater because of its high efficiency, low cost, and wide adaptability [65]. Numerous absorbents, such as agricultural and industrial solid waste, biomaterials, nanostructured materials, and porous structure materials, have been used for wastewater adsorption treatment. Dyes and heavy metal pollutions are one of the most critical environmental concerns, and it is crucial to purify and remove them prior to releasing them into water resources. Researchers are becoming more and more concerned about how to get rid of these high-toxicity pollutants, even when they are only in small amounts. The high surface area and active adsorption sites provided by aerogels (Figure 6), in addition to their natural biopolymer composition (e.g., cellulose and chitosan), make them compelling candidates for water-pollutant removal [92,93].

### 4.1. Mechanisms of Water Treatment Using Nanocellulose Materials

Various methods can remove heavy metal ions and dyes from water and industrial wastewater. These methods can be grouped into three categories: physical, chemical, and biological [94]. Physical methods that use processes such as coagulation, flocculation, adsorption, filtration, and sedimentation are simple and cost-effective but may not remove all types of contaminants and create waste. Chemical methods that use reactions such as precipitation, oxidation, and ozonization can remove a wide range of contaminants but can be expensive and require hazardous chemicals. Biological methods that use microorganisms or plants are environmentally friendly and remove organic contaminants but may not remove certain contaminants and need careful monitoring. Among all the methods available, adsorption is a promising method for the removal of pollutants from wastewater, with the potential to offer economic and environmentally friendly solutions for treating industrial effluents [94]. It is a process used to separate certain components from a fluid phase by attracting them to the surface of a solid adsorbent. The adsorbate, which may be ions, atoms, or molecules, is transferred and adhered to the surface of the adsorbent by physical forces or chemical bond formations. Adsorption has been effectively used to remove heavy metals and dyes from wastewater, offering advantages over conventional methods such as low operating costs, minimal sludge generation, high detoxification efficiency, and no nutrient requirements. Moreover, adsorption does not produce harmful substances.

**Figure 7:**
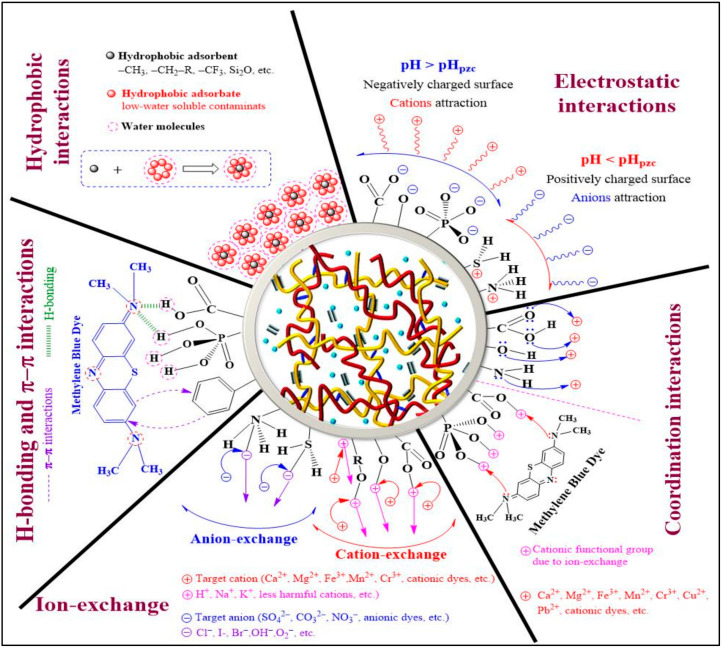
Possible adsorbent–adsorbate interaction mechanisms for the decontamination of wastewater using a cellulose hydrogel or aerogel. Adapted from [98].

Understanding the adsorption process and mechanisms is crucial to improving their performance in removing pollutants. The adsorption process depends on factors such as functional groups, adsorbent properties, pollutant composition, and experimental parameters [95]. According to several studies, the introduction of extra functional groups, including phosphonate, carboxyl, and sulfonate groups, to cellulose can improve the adsorption of heavy metal ions [96]. This is because these groups increase the electronegativity of the adsorbent surface. Figure 7 illustrates the various adsorption mechanisms for dyes and heavy metals on aerogels. Electrostatic interaction is when charged molecules interact with each other, either attracting (oppositely charged—cation–anion interactions) or repelling (similarly charged—cation–cation or anion–anion interactions) each other [97]. To remove ionic pollutants using certain materials, those materials must have a charge opposite to the ions that need to be removed. Cellulose aerogel can be made with specific functional groups that produce opposite charges in the target ions [95]. The pH of the solution also affects the charge of the adsorbent surface, with different functional groups being protonated or deprotonated depending on the pH. This can create attractive interactions between oppositely charged ions and the adsorbent surface. Studies have shown that electrostatic interactions are typically the primary mechanism in the removal of pollutants such as heavy metals and dyes from aqueous solutions [98].

Hydrogen bonding is a type of dipole–dipole interaction between a positively charged hydrogen atom and a more electronegative atom (e.g., N, O, F, etc.) or group. It plays a crucial role in treating dye-containing wastewater, where functional groups in the adsorbent molecule participate in hydrogen bonding with pollutants. Researchers have developed eco-friendly CMC nanofiber hydrogels that can remove several chemical dyes via hydrogen bonding, electrostatic interactions, and hydrophobic interactions [99]. Hydrogen bonding interactions between sulfur atoms in both anionic (acid blue 93) and cationic (methylene blue) dyes and hydrogen atoms in aerogel material have been extensively explained [100]. Therefore, hydrogen bonding offers promising opportunities for developing effective adsorbents to remove pollutants from wastewater.

Ion exchange is a commonly used process in the treatment of wastewater, as it provides an effective means of removing unwanted dissolved ions such as cations or anions from an aqueous solution. The process involves an exchange of ions between a liquid (wastewater) and an insoluble solid (adsorbent), where the unwanted dissolved ions are removed and replaced with ions of the same charge on the adsorbent surface. In a perfect ion exchange process, the number of ions released from the adsorbent surface is equivalent to the number of ions adsorbed by the adsorbent molecules [98]. This process is highly effective for the removal of hazardous pollutants such as dyes and heavy metals from wastewater. By transforming pollutants into a shape in which they can be recycled, this process decreases the degree of hazardous load, leaving behind less harmful elements in their place or enabling ultimate discharge by decreasing the hydraulic flow of the stream carrying toxic elements.

A hydrophobic interaction occurs between non-polar compounds (hydrophobes) and water molecules, which are weakly attracted to each other because of van der Waals forces [101]. This interaction is utilized in wastewater treatment to remove non-polar pollutants such as pigments, disperse dyes, and organic compounds. Hydrophobic interactions play an essential role in engineering the mechanical properties of cellulose-based aerogels and are used as physical crosslinking points during chemical reactions [102]. Hydrophobic interactions are also involved in adsorbing insoluble organic pollutants into cellulose cryogels. However, cellulose-based hydrogels are often modified with both hydrophilic and hydrophobic functional groups to remove both soluble ionic pollutants and water-insoluble contaminants.

The π–π interaction is a non-covalent interaction between adsorbent and adsorbate molecules in an aqueous solution. It affects various chemical properties and depends on the functional groups present on both surfaces and the pH of the solution [103]. Different types of π–π interactions can be formed based on electron-donor or electron-acceptor behavior. The π–π interaction occurs when one of the molecules involved has an electron-rich or deficient group in the structure of benzene or other aromatic rings, which results in interactions in an aqueous medium [98]. The nature of the π–π interaction depends largely on the functional groups present on the adsorbent and adsorbate surfaces, as well as the pH of the solution. These interactions are commonly found in the adsorption of pollutants and dyes onto graphene-, graphene-oxide-, or carbon-based aerogels. π–π stacking is reported as the primary driving force in the removal of heavy metal ions and organic dyes. The role of GO in hydrogel or aerogel is to enhance mechanical strength and adsorption capability. Adsorption mechanisms for dyes involve both electrostatic and π–π interactions, while those for heavy metals involve electrostatic interactions, surface complexation, and ion exchange [104].

The coordination interaction involves the sharing of both electrons by a single atom, and it plays a crucial role in the removal of cations and dyes from wastewater through adsorption mechanisms. In this process, cations are attracted to functional groups on the adsorbate surface that contain lone-pair electrons, such as O and N. Coordination interactions often occur in combination with other interactions such as ion exchange and electrostatic interactions. For instance, in the removal of Cu^2+^ using sugar cane bagasse cellulose and gelatin-based composite hydrogels, Cu^2+^ ions form coordination bonds with N or O atoms sourced from –NH_2_ and –OH functional groups, respectively [105]. Similarly, coordination interactions occur between metal ions and O atoms (from –OH groups) during the removal of Hg^2+^, Pb^2+^, and Cu^2+^ using a biopolymer composite adsorbent [106]. The adsorption mechanism of chitosan/cellulose composite adsorbent for the removal of Congo red (CR) dye involves electron sharing (coordination interaction) and transfer (electrostatic adsorption) between the adsorbent and adsorbate molecules [107].

### 4.2. Removal of Organic Dyes

Synthetic dyes are widely used in various industries such as textiles, paper, leather, food, and cosmetics. However, organic dye molecules can strongly bind to metal ions, making them toxic to aquatic organisms. Additionally, dyes in waterbodies can reduce photosynthetic activities by blocking light penetration, inhibiting the growth of aquatic flora [108]. For example, malachite green (MG) is a cationic dye that has been widely used in aquaculture as a parasiticide, fungicide, and antiprotozoan, as well as in medical disinfectants. Therefore, because of the harmful effects of MG accumulation in living organisms, such as cytotoxicity, mutagenicity, teratogenicity, and carcinogenicity, it is crucial to remove this dye from wastewater. Among various methods for removing dyes, adsorption is one of the most effective and widely used techniques, along with sedimentation, filtration, chemical coagulation, oxidation, or treatment with microorganisms. The use of adsorption materials is promising because they have been found to be effective not only for removing dyes and oils but also other organic contaminants such as antibiotics from polluted water [2].

In this context, various studies have explored the use of cellulose nanofiber to develop adsorption material (Table 3). For example, Liu and colleagues introduced a novel cellulose adsorbent that was modified with acrylamide and acrylic acid in three dimensions to eliminate dye pollutants from aqueous solutions [100]. This adsorbent was found to be effective in removing both anionic dye acid blue 93 and cationic dye methylene blue from single or binary dye water solutions. The adsorbent also exhibited good recyclability. Table 2 summarizes some of the recent dye-removal studies carried out using cellulose aerogels.

Further, Jiang and co-workers have developed an ultralightweight aerogel from TEMPO-oxidized CNFs through a three-step process involving freezing–thawing–induced hydrogel formation, tert-butanol exchange, and freeze-drying to create a honeycomb cellular structure consisting of irregularly shaped open cells surrounded by mesoporous thin walls of self-assembled CNFs [112]. This aerogel exhibited a high specific surface area (193 m^2^/g) and a negatively charged surface that facilitated the removal of cationic dyes. The MG adsorption on CNF aerogels showed a monolayer Langmuir adsorption isotherm with a maximum adsorption capacity of 212.7 mg/g, corresponding to the participation of 46% of the total carboxyl’s surface. The CNF aerogel showed over 92% MG removal efficiency in single-batch adsorption at a 10:5 mg/mL aerogel/MG ratio and a 100 mg/L dye concentration. Ren and co-workers reported that using an RCE/GO composite aerogel resulted in uniformly stacked lamellar pore structures after the addition of GO [114]. Static adsorption tests revealed that the MG adsorption rate of the composite aerogel was nearly 340% higher than that of the RCE aerogel. 

Generally, the adsorption capacity of an adsorbent is governed by the source and the surface properties, such as porosity and surface area [120]. Among the many adsorbents listed in Table 2, adsorbents with higher surface properties such as higher micropore volume, smaller pore size, and higher surface area showed higher adsorption capacities. Adsorption technologies have been extensively studied for dye removal, and cellulose-based carbon aerogels have exhibited the highest adsorption capacity of 1947 mg/g for malachite green, mainly because of their improved surface properties, including reduced micropore diameter, increased micropore volume, and relatively large surface area [115]. However, adsorption technologies have several drawbacks where the dye adsorption mechanism depends mainly on the type of dye. 

### 4.3. Adsorption of Heavy Metal

In recent years, wastewater containing heavy metal ions has increased, posing a long-term threat to human health and natural ecosystems because of their severe toxicity, non-biodegradability, and tendency to accumulate in the body via the food chain. Ion exchange, coagulation–precipitation, membrane filtration, reverse osmosis, adsorption, and others have been developed to efficiently remove heavy metal ions [2]. However, industrial wastewater doped with Cr^2+^, Cu^2+^, and Co^2+^ ions requires expensive electrochemical treatment, which violates energy-saving and green production principles. Current research is focusing on removing these ions from water using adsorption materials such as aerogels and their derivatives.

According to various studies, cellulose aerogels have a remarkable effect on the adsorption of heavy metal ions. For the removal of Cu^2+^, cellulose aerogels have been combined with other materials such as graphene oxide, waste paper, UiO-66-NH, polyethyleneimine, and polydopamine [117,121,122,123,124]. Mixing with additional materials improves the physical features of the resulting aerogel while simultaneously increasing its adsorption efficiency [65]. Other materials, such as polyethyleneimine, graphene, EDTA, β-Cyclodextrin, ferrocenecarboxaldehyde, polydopamine, hexamethylenetetramine, and zeolite, have also been reported to increase the adsorption of heavy metal ions such as Cr (VI), iodine 129, cesium, Hg^2+^, Cd^2+^, phosphate, and Pb (II) [48,123,124,125,126,127,128,129,130,131,132,133]. Table 4 summarizes the heavy metal ions adsorbed by different cellulose-based aerogels.

Different aerogel formulations have different properties, including their surface areas, densities, structures, and efficiencies. In addition, there are several parameters that influence the adsorption efficiency of the aerogel used, such as pH and temperature [2]. Despite numerous works in this research direction, the suitability of cellulose-based aerogel in heavy metal and dye removal from an aqueous medium is mainly limited to laboratory-based batch experimental results and very few in continuous mode operation.

### 4.4. Reusability

The regeneration and reuse of adsorbents are essential aspects that greatly contribute to reducing the overall cost of the adsorption process. The ability to reuse adsorbents is a significant factor when considering their practical application, as it leads to increased efficiency and cost reduction cost [148]. Numerous studies have focused on demonstrating the reusability of cellulose aerogel in effectively removing organic dyes and heavy metals.

One such study conducted by Liang and colleagues explored the reusability of cellulose aerogel and its exceptional activity in removing organic dyes across five cycles of reuse [149]. Similarly, Lei and co-workers reported similar findings regarding the reusability of cellulose aerogel for the removal of heavy metals [123]. Other research reported that the regenerated cellulose aerogel exhibited highly efficient pollutant adsorption, with an efficiency of over 97% for three regeneration cycles. However, with further regeneration and recycling, there was a slight reduction in efficiency to approximately 95% [150].

The slight decline in pollutant adsorption performance observed during subsequent cycles can be attributed to the presence of preabsorbed pollutant particles or molecules that become trapped within the aerogel structure. This phenomenon reduces the overall availability of negatively charged carboxyl groups, which are crucial for effective adsorption in subsequent cycles [151].

## 5. Conclusions

Despite showing promising outcomes, the advancement of aerogels faces various challenges that need to be addressed before they can be introduced to the end market. One of the primary concerns is the material science of adsorbents, which should offer superior performance in terms of fast reaction, reliability, ease of use, reuse, and final recyclability compared with existing solutions. To achieve this, several aims must be considered in the processing of aerogels, including the use of novel raw materials, procedures with appropriate resource management, and competitive production costs. This requires a detailed investigation of aerogel supplies from renewable and non-renewable sources, including waste and by-products. The success of aerogel production also depends on the implementation of best practices, facilities, and experience, which are essential for lab-to-industrial scale-ups. To bring aerogel advances to the market, multidisciplinary teams need to work together to transform these advances into scientific, technological, and regulatory improvements. Additionally, to achieve superior performance in aerogels, there is a need to test and optimize new formulations that combine different materials, such as polymers and metal oxides, which can lead to useful devices with specified features in the final system. 

Polysaccharide-based aerogels, especially cellulose and chitosan aerogels, have garnered attention in recent years for their use in environmental applications, including the removal of pollutants such as dyes and heavy metals. Because of their large surface area and sorbent ability, these materials are effective in removing these pollutants from wastewater. Moreover, the mechanical resistance, softness, and responsiveness of these materials make them a great choice for numerous environmental applications. For example, polysaccharide-based aerogels have been used for the removal of heavy metals such as lead, cadmium, and chromium from wastewater. Additionally, they have been used in the removal of synthetic dyes from industrial wastewater, which can cause significant harm to the environment if not treated properly. The utilization of polysaccharide-based aerogels offers a promising solution for effectively eliminating pollutants from water sources. This approach not only enhances the overall quality of our water resources but also mitigates the detrimental impacts of pollution on the environment. Furthermore, the use of biopolymers that are biocompatible opens up the possibility of applying these aerogels to various other fields such as food, packaging, biomedical applications, and many more. This versatility expands the potential benefits of employing polysaccharide-based aerogels, making them a cost-effective and environmentally friendly solution with wide-ranging applications.

## Figures and Tables

**Table 1:** Illustration of the effects of commonly used dyes in textile industries on human health.

Dye	Heath Effect	Ref.
Malachite green	Carcinogenic and can have serious effects on human reproductive and immune systems.	[71]
Disperse blue 291	Can cause DNA fragmentation and increase the apoptotic index in humans.	[72]
Disperse red 1	Mutagenic and can increase the frequency of micronuclei, indicating mutagenic activity at the chromosome level.	[70]
Disperse orange 1	Causes DNA damage through base-pair substitution and frameshift mutations; cytotoxic; induces apoptosis.	[73]
Methylene blue	Harmful to human health and can cause various problems, including breathing trouble, stomach problems, blindness, mental and digestive issues, skin/eye irritation, and even the death of cells in tissues.	[74]
Crystal violet	Has toxic effects as a mitotic poison and carcinogen; potentially causes damage to chromosomes and promotes tumor growth. Moderate eye irritation, sensitivity to light, and permanent damage to the cornea and conjunctiva.	[75]
Congo red, basic red	Impact on blood factors, including blood clotting; can cause irritation to the eyes, skin, and digestive system; may trigger allergic reactions, drowsiness, and breathing difficulties. Metabolizes into benzidine, a carcinogenic and mutagenic substance in humans.	[76]
Methyl orange/ orange 3	Water-soluble azo dyes are recognized as being carcinogenic and acidic or anionic. Can cause vomiting and diarrhea, and exposure to high levels can be fatal.	[77]
Remazol brilliant blue/reactive blue 19	Reported as the main cause of bladder cancer in humans, splenic sarcomas, hepatocarcinoma, and chromosomal aberrations in mammalian cells.	[78]
Rhodamine B	Harmful if consumed by humans and animals. Can result in skin, eye, and respiratory tract irritation; kidney disease; and cancer. Reduces light penetration and decreases the rate of photosynthesis and respiration.	[79,80]
Metanil yellow	Causes harm to several vital organs and organ systems in humans, enhances tumor growth, and causes damage to the testicles leading to a cessation of sperm production in guinea pigs. Linked to hematological effects, disruptions in DNA synthesis, and cases of allergic contact dermatitis	[81]

**Table 2:** A summary of the effects of heavy metal ions on human health and their sources.

Heavy Metal Ions	Toxic Effects	Source	Ref.
Ag	Discoloration of skin, respiratory problems	Mining of copper, copper–nickel, lead, and lead–zinc ores	[85,86]
As	Cancer, skin conditions, stomach pains	Arsenic mining, smelting coal combustion, industrial production	[86,87]
Cd	Carcinogenic, mutagenic, endocrine disruptor, kidney and bone damage	Rainwater and surface waters in industrialized and urban zones, bedrock erosion or weathering, leakage from polluted locations and landfills	[86,87,88]
Cr	Hair loss, headache, diarrhea, nausea, increased cancer risk	Tanneries, pulp and rubber manufacturing processes, metallurgical industries	[86,87,88]
Cu	Brain and kidney damage, liver cirrhosis, chronic anemia, insomnia	Copper mining, smelting of metal, mineral processing, production of steel, electroplating, etching, production of plastics	[87,88,89]
Hg	Autoimmune diseases, brain and lung damage, rheumatoid arthritis, circulatory and nervous disorders	Industrial activity, electronic industry, mining	[85,86,87]
Ni	Allergic skin diseases; cancer of the lungs, nose, sinuses, and throat; hair loss; chronic asthma	Wind-blown dust, residual and fuel oils, municipal waste incineration, nickel mining and refining	[86,87,90]
Pb	Impaired development, reduced intelligence, coordination problems, increased risk of cardiovascular disease	Road transport, wastewater from lead batteries, ammunition, glass and ceramic industry, sewage from lead chemicals, electronic waste, mining production	[86,87,88]
Zn	Dizziness, fatigue, anemia, decreased immune function	Industrial and agricultural activities	[86,87]

**Table 3:** Adsorption of industrial dyes using cellulose-based adsorbents.

Adsorbents	Dyes	Maximum Adsorption Capacity, Qm (mg/g)	Ref.
Cellulose aerogel modified with acrylamide and acrylic acid	Acid blue and methylene blue	1372	[100]
CNC-COOH (TEMPO)	Methylene blue	769	[109]
CNC-NH2 (oxidation with NaIO_4_ followed by reaction with ethylenediamine)	Acid red	556	[110]
CNC-NH2 (grafted with PVAm)	Acid red Congo red Light yellow	869 1469 1250	[111]
CNF-COOH (TEMPO)	Malachite green (MG)	212.7	[112]
CNC-reinforced keratin aerogel	Rhodamine B Congo red	1201 1070	[113]
Graphene oxide (GO) and regenerated cellulose (RCE) composite aerogel	Methylene blue	68	[114]
Cellulose-based carbon aerogels	MG MB	1947 1192	[115]
Cellulose nanofibril aerogels	MG	212.7	[116]
PEI-crosslinked PDA-CNF	Methyl orange	265.9	[117]
Carboxymethyl cellulose/reduced graphene oxide aerogel (CMCrGA)	Rhodamine B	161.29	[118]
Cellulose nanoparticle–graphene oxide aerogels	Methylene blue	111.2	[93]
N-doped carboxymethyl cellulose-based carbon aerogels	Methylene blue (MB) Congo red TRB G133	230.4 85.2 73.3	[119]

**Table 4:** Heavy metal removal using different types of cellulose-based aerogels.

Heavy Metal Removed	Aerogel Type	Surface Area (SA)/Pore Diameter (PD)/Porosity/Pore Volume (PV)/Density (D)/Remarks	Structure	Conditions	qmax (mg/g)	Ref.
Cd^2+^	Bacterial cellulose (BC)/poly(amidoxime) (PAO) aerogel	-	-	Dosage = 0.2 g/L pH = 6 Co = 10 ppm	382.3	[134]
	Multi-crosslinked chitosan hydrogels and aerogels	-	-	Temp = −80 °C Time = 48 h	99.75	[135]
Cr(VI)	Polyethyleneimine (PEI)-grafted adsorbent, a cellulose@PEI aerogel (CPA-2)	SA = 36.77 m^2^/g PD = 13.5 nm	When the hydrogels were modified with PEI molecules, a good mesoporous structure was obtained in a three-dimensional network of composites.	Co = 100 mg/L	96.8	[136]
	Superparamagnetic γ-Fe_2_O_3_ nanoparticles encapsulated in three-dimensional architectures of cellulose aerogels	SA = 136.8–173.5 m^2^/g PD = 3–140 nm PV = 0.88–1.02 cm^3^/g	The aerogels were able to obtain a three-dimensional skeleton structure that provided greater resilience against shrinkage and collapse during the preparation process.	pH = 3 Dosage = 2 mg/L Contact time = 2 h T = Room temperature Co = 10 mg/L	10.2	[137]
Cu^2+^	MnFe_2_O_4_–cellulose aerogel composite	SA = 229–288 m^2^/g PD = 13.33–13.99 nm PV = 0.67–0.88 cm^3^/g D = 0.1021–0.2087 mg/cm^3^	The introduction of FeCl_3_/MnCl_2_ particles resulted in a 3D porous network with a significant impact on the physical characteristics.	Dosage = 2 mg/L Co = 1–250 mg/L T = 25 °C Contact time = 2 h pH = 6	63.3	[138]
	TA@CNF-CDA aerogel	SA = 75.66–150.61 m^2^/g PD = 4.133–3.822 nm PV = 0.047–0.012 cc/g	The 3D porous structure was characterized by thin sheets and honeycomb-like pores. The surface exhibited broadened fibrils and dark shadows.	Co = 50 mg/L pH = 5	45.5	[139]
	Amide-functionalized cellulose-based	PD > 200 μm	A well-arranged skeleton with a uniform dispersion of sizable pores.	pH = 7	59.88	[140]
	Carboxylated cellulose cryogel beads	-	-	pH = 5.6 Dosage = 2 mg/L T = 25 °C Co = 50–400 ppm	84.12	[141]
Hg^2+^	TEMPO-oxidized (TO) nanofibrillated cellulose (TO-NFC) aerogel	SA = 43.57 m^2^/g D = 1269 kg/m^3^ The porosity was 99.10%. A decrease was noticed in porosity after adsorption	The aerogels exhibited an interlinked porous structure composed of multiple thin sheets, possessing remarkable shape recovery and flexibility. Compared with the TO-NFC aerogel, the surface texture of the TO-NFC-Si-SH aerogel was rougher.	Co = 1–410 mg/L pH = 7 T = 25 °C	140.25	[142]
Pb^2+^	Bentonite-modified chitosan/microcrystalline cellulose aerogel-prepared	SA = 99.1573 m^2^/g PD = 176–228 μm and 3–7 nm	An adsorbent with a lightweight and rough surface, featuring a honeycomb briquette structure.	Co = 90 mg/L	116.54	[143]
	Bacterial cellulose graphene oxide composite	SA = 21.58–49.99 m^2^/g PD = 25.388 Å PV = 0.0356–0.0307 m^3^/g	The introduction of graphene oxide resulted in the production of a mesoporous green aerogel, which exhibited significant changes in its physical characteristics.	Dosage = 5 mg Contact time = 28–30 min T = 25 °C and 40 °C Co = 60 mg/L pH =6	303.03	[144]
	Bacterial cellulose/polyvinyl alcohol/ graphene oxide/attapulgite (BC/PVA/GO/AP)	SA = 47.35–8.47 m^2^/g PV = 0.215–0.027 m^3^/g	An aerogel with a porous structure characterized by irregular and flat slit shapes, along with cracks or wedges on its surface.	-	217.8	[145]
	CNFs crosslinked with acrylic acid (AA)-CA aerogels	The pore size of the CA aerogel was several micrometers	The CNF had a diameter ranging from 5 to 50 nm. The CA aerogel possessed a porous structure that remained well-preserved after the process of adsorption.	Co = 200 mg/L pH = 5.6 T = 303 K	137.741	[146]
U(VI) (static adsorption)	TEMPO-oxidized CNF aerogel	SA = 187 m^2^/g	A strong and durable three-dimensional configuration consisting of slit-shaped tubular capillary pores.	Dosage = 0.05 g Co = 5–50 mg/L pH = 5	440.6	[147]
Zn^2+^	Bacterial cellulose (BC)/ poly(amidoxime) (PAO) aerogel	SA = 428 m^2^/g PD = 0.58 cm^3^/g D = 1.543 mg/cm^3^	The material exhibited a sponge-like network composed of interconnected sheet-like structures, with numerous 3D microscale porous channels ranging in width from 0.01 to 0.1 μm.	Dosage = 0.2 g/L pH = 6 Co = 10 ppm	494	[134]

## Data Availability

Not applicable.
